# Acute liver failure due to herpes simplex viral hepatitis diagnosed by skin lesions and blood tests: a case report

**DOI:** 10.1186/s13256-023-04083-w

**Published:** 2023-08-10

**Authors:** Kento Shionoya, Makoto Kako, Sakue Masuda, Makomo Makazu, Kazuya Koizumi

**Affiliations:** Shonan Gastroenterology Medicine Center, Shonan Kamakura General Hospital, Okamoto 1370-1, Kamakura-Shi, Kanagawa, 247-8533 Japan

**Keywords:** Herpes simplex viral hepatitis, Acute liver failure, Acyclovir, Plasma exchange, Hemodiafiltration, Case report

## Abstract

**Background:**

The incidence of acute liver failure from herpes simplex virus is rare.

**Case presentation:**

A 71-year-old Japanese man was diagnosed with acute liver failure and was transferred to our hospital. Steroid therapy, plasma exchange, and hemodiafiltration were started for liver failure, and antimicrobial therapy was initiated for pneumonia. *Staphylococcus epidermidis* was detected in blood culture. Skin rash appeared; a positive anti-herpes simplex virus result led to the diagnosis of acute liver failure from herpes simplex virus. Hence, acyclovir was started. After blood tests improved, treatments for acute liver failure were discontinued. Antimicrobial therapy was continued; however, he died. In this case, persistent bacteremia and drug-induced liver damage due to acyclovir may have contributed to his death.

**Conclusions:**

Acute liver failure can lead to complications and death. Thus, careful observation is crucial, even if the patient has shown some improvements.

## Background

Herpes simplex virus (HSV) infection manifests with a variety of symptoms. Although it is known that HSV infections cause liver dysfunction, acute hepatitis caused by HSV is rare. Acute hepatitis has been noted in 2–4% of cases of viral hepatitis and in 0.8% of cases of acute liver failure [1, 2]. Acute liver failure has a high mortality rate, and the risk of developing it is increased in patients with underlying diseases such as cancer and human immunodeficiency virus infection; however, it may occur also in people with normal immune function. Herein, we report a case of acute liver failure due to HSV hepatitis.

## Case presentation

**Fig. 1:**
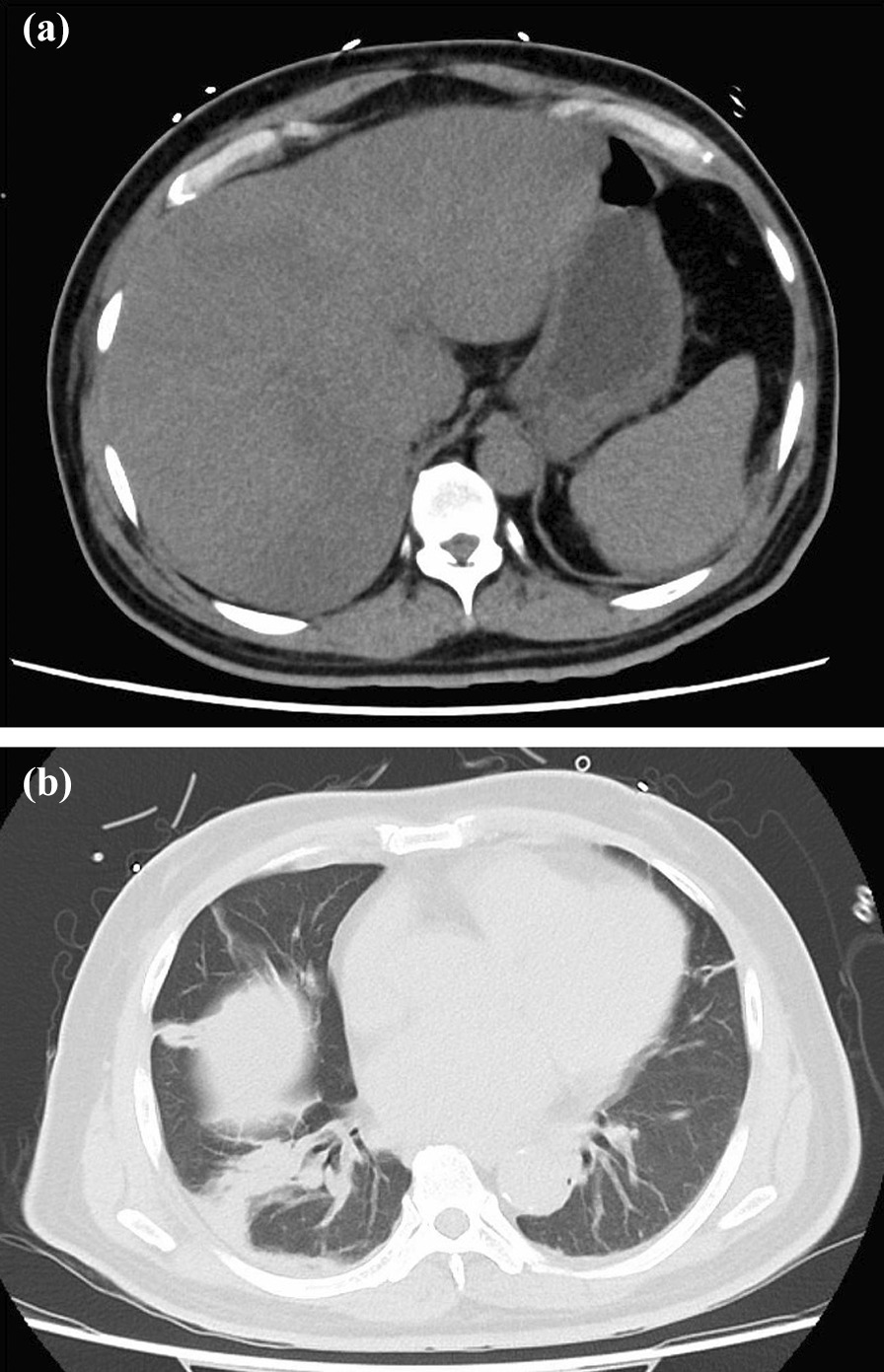
Plain chest and abdominal computed tomography scans were obtained on the day of transfer to our hospital. **a** Low absorbance of the liver, hepatomegaly, edematous thickening of the gallbladder, and splenomegaly are noted. **b** An infiltrative shadow on right lung is noted, which was diagnosed as pneumonia

**Fig. 2:**
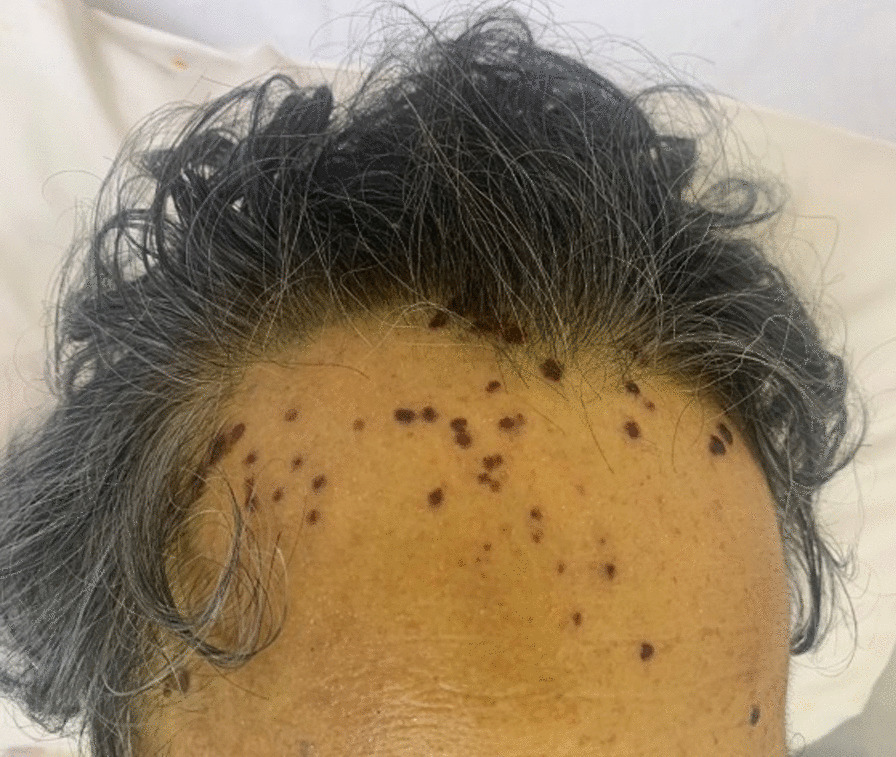
Skin lesions on the face. Numerous small blisters appeared, mainly on the forehead

**Fig. 3:**
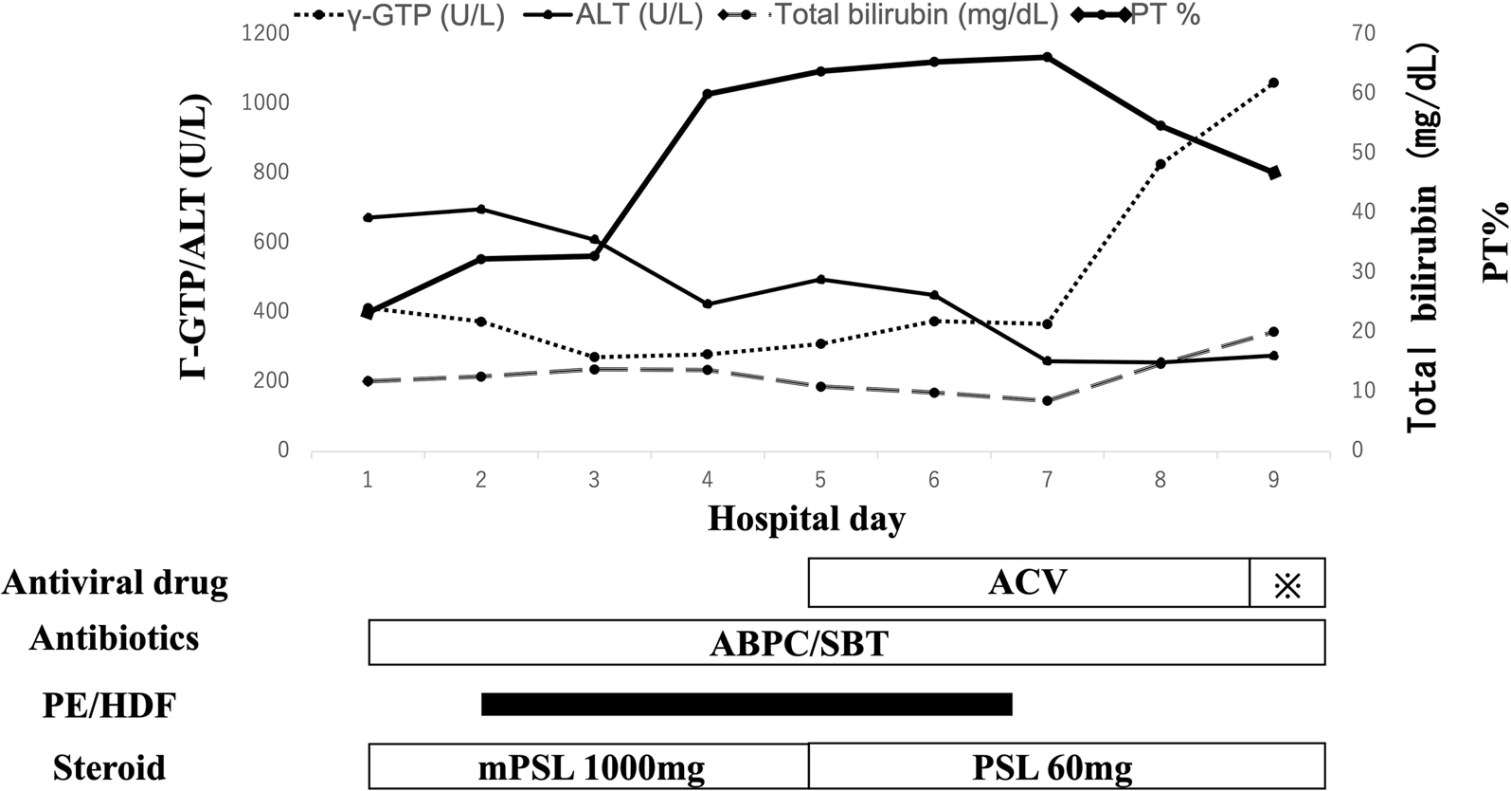
The patient’s clinical course. *γ-GTP* γ-glutamyl transferase, *ABPC/SBT* ampicillin/sulbactam, *ACV* acyclovir, *ALT* alanine aminotransferase, *HDF* hemodiafiltration, *mPSL* methylprednisolone, *PE* perform plasma exchange, *PSL* prednisolone. ※, amenavir

A 71-year-old Japanese man visited a hospital due to general weakness, persistent fever, and difficulty in drinking water and swallowing 3 days before the visit. The patient had a history of chronic heart failure and atrial fibrillation. Laboratory test results revealed elevated levels of hepatobiliary enzymes [aspartate aminotransferase (AST), 609 U/L; alanine aminotransferase (ALT), 488 U/L; γ-GTP, 407 U/L; lactate dehydrogenase (LDH), 1219 IU/L; total bilirubin, 6.3 mg/dL], a normal white blood cell (WBC) count of 6200/mm^3^, a C-reactive protein level of 32.93 mg/dL, and coagulation abnormalities [prothrombin time (PT), 36.0 seconds; percentage of prothrombin (PT%), 17.6%; prothrombin time–international normalized ratio (PT–INR), 3.94; and activated partial thromboplastin time (APTT), 52.0 seconds]. The patient’s laboratory test results are presented in Table 1. MELD score was 32. He was diagnosed with acute hepatitis and admitted. He was kept on bed rest and started on intravenous fluids. The following day, his blood test results showed a significant increase in the levels of hepatobiliary enzymes (AST, 915 U/L; ALT, 570 U/L; γ-GTP, 407 U/L; LDH 1365 IU/L; and total bilirubin, 9.4 mg/dL). In addition, the platelet count decreased to 8.5 × 10^4^/μL, and the PT% increased to 23.7%. Acute liver failure was considered, and he was transferred to our hospital and admitted to the intensive care unit. Plain chest and abdominal computed tomography showed hepatomegaly with diffuse low attenuation, edematous thickening of the gallbladder, and splenomegaly (Fig. 1a), which suggested acute hepatitis. Hepatitis B surface antigen and hepatitis C virus antibody results were negative. He had no history of drinking alcohol, and there was no recent ingestion of raw food or initiation of new medications. Hence, the possible cause of liver dysfunction was unclear. A liver transplant was considered and discussed with a transplant facility; however, due to his age, a transplant was not feasible. It was determined that early intervention should instead be performed. We initiated steroid pulse therapy with 1000 mg/day of methylprednisolone. To treat hyperammonemia, lactulose, rifaximin, and probiotics were started. Additionally, he had pneumonia and was started on ampicillin/sulbactam (Fig. 1b). Plasma exchange (PE) and hemodiafiltration (HDF) were initiated on the second day of hospitalization. In HDF, 450 mL of dialysate was supplied per minute and 250 mL was used as replacement fluid per minute. However, his ammonia level gradually worsened, and his level of consciousness deteriorated. The oxygen demand increased due to hepatopulmonary syndrome. A nasal high-flow cannula was attached; however, his respiratory condition and consciousness further worsened. On hospitalization day 3, the patient underwent intubation and mechanical ventilation. Although liver failure did not improve, PE and HDF were continued. Additionally, *Staphylococcus epidermidis* was detected in the blood culture in all four bottles collected taken at the time of admission, and a diagnosis of bacteremia was made. On hospitalization day 5, a skin rash appeared on his face, which suggested HSV infection (Fig. 2). At this time, anti-HSV IgM antibody was detected from blood specimens collected at the time of hospitalization. Although we were unable to perform a liver biopsy because of marked coagulation abnormalities, no other factors that could have caused liver damage were observed, and we diagnosed the condition as HSV hepatitis. Acyclovir (ACV) was then initiated, and 1000 mg/day of mPSL was administered for 5 days, followed by 60 mg/day of prednisolone as post-therapy. After initiating ACV, blood test results showed that his coagulation parameters, ammonia levels, and bilirubin levels had improved. Hence, PE and HDF were discontinued on hospitalization day 6. Furthermore, his level of consciousness improved, and he was weaned from mechanical ventilation; however, fever and elevated inflammatory markers persisted. Acute liver failure eventually resolved. However, levels of hepatobiliary enzymes increased, and drug-induced liver injury of the bile congestion type due to ACV was suspected; hence, ACV was discontinued. We replaced ACV with oral amenavir on hospital day 9; however, his condition further deteriorated, and he died on the same day. As consent for a pathological autopsy could not be obtained, histological analysis of the liver could not be performed (Fig. 3).

**Table 1:** Laboratory data on the day of the transfer to our hospital

Biochemistry		Na	127 mmol/L	Immunological tests	
AST	1049 IU/L	K	4.6 mmol/L	HBc Ab	(−)
ALT	673 IU/L	Cl	93 mmol/L	HBs Ag	(−)
LDH	1437 IU/L	Ca	7.3 mg/dL	HBs Ab	(+)
γ-GTP	413 IU/L	IP	3.5 mg/dL	HCV Ab	(−)
ChE	158 IU/L	Blood cell count		ANA	(−)
Cr	1.82 mg/dL	WBC	6900/μL	IgM-HAV	(−)
BUN	43.1 mg/dL	Hb	16.6 g/dL	HSV-IgM	(+)
T-Bil	11.8 mg/dL	Plt	78,000/μL	HSV-IgG	(+)
D-Bil	8 mg/dL	Coagulation tests		VZV-IgM	(−)
CK	85 IU/L	PT	29.3 seconds	VZV-IgG	(+)
Alb	2.6 mg/dL	PT%	23.3	EBV-IgM	(−)
Fe	56 μg/dL	PT–INR	2.62	EBV-IgG	(+)
Ammonia	118 μg/dL	APTT	46.5	CMV-IgM	(−)
Ferritin	14,009 ng/mL	D-dimer	19.7 μg/mL	CMV-IgG	(+)
CRP	29.48 mg/dL	Fib	602.3 mg/dL	IgA-HEV	(−)
		FDP	34.3 μg/dL	AMA	(−)
		AT-III	44.1	HSV-PCR	1000/μg DNA

*γ-GTP* γ-glutamyl transferase, *Alb* albumin, *ALP* alkaline phosphatase, *ALT* alanine aminotransferase, *AMA* anti-mitochondrial antibody, *APTT* activated partial thromboplastin time, *AST* aspartate aminotransferase, *AT-III* antithrombin-III, *BUN* blood urea nitrogen, *Ca* calcium, *ChE* cholinesterase, *CK* creatine kinase, *Cl* chloride, *Cre* creatinine, *CRP* C-reactive protein, *D-Bil* direct bilirubin, *EBV* Epstein–Barr virus, *FDP* fibrinogen degradation product, *Fe* iron, *Hb* hemoglobin, *HBcAb* hepatitis B core antibody, *HBsAb* hepatitis B surface antibody, *HBsAg* hepatitis B surface antigen, *HCV Ab* hepatitis C virus antibody, *Ht* hematocrit, *Ig* immunoglobulin, *IP* inorganic phosphorus, *K* potassium, *LDH* lactate dehydrogenase, *Na* sodium, *Plt* platelet count, *PT* prothrombin time, *PT%* percentage of prothrombin, *PT–INR* prothrombin time–international normalized ratio, *RBC* red blood cell count, *T-Bil* total bilirubin, *TP* total protein, *VZV* varicella-zoster virus, *WBC* white blood cell count

## Discussion and conclusion

We report a rare case of acute liver failure due to HSV hepatitis diagnosed by skin lesions, elevated levels of anti-HSV IgM antibody, and presence of HSV-DNA in blood by polymerase chain reaction (PCR) test. Post-mortem examination confirmed HSV-DNA positivity. Acute liver failure has a high mortality rate and has various causes, including viruses and drugs [3, 4]. Steroid use, human immunodeficiency virus infection, cancer, renal failure, and pregnancy increase the risk of HSV hepatitis, and the disease is known to cause early death after liver transplantation [5–7]. As shown in this case, HSV hepatitis may occur even in people with normal immune function [8]. In immunocompromised patients, the risk of mortality is high, even when they are treated with parenteral antiviral agents [5, 7, 9]. As symptoms of HSV hepatitis are nonspecific, it is difficult to diagnose the disease. In previously reported cases, clinical findings in HSV hepatitis included fever, coagulation abnormalities, encephalitis, and acute renal failure. Additionally, a skin eruption specific to HSV infection, which consists of a large number of painful small blisters, mainly on the forehead, develops in about half of patients [10, 11]. Furthermore, HSV hepatitis may present with a high fever, elevated levels of hepatic enzymes, and a significantly decreased WBC count [11]. Due to its nonspecific symptoms and rarity, HSV hepatitis is often a delayed or missed diagnosis, leading to treatment delays that result in a poor prognosis. Screening for IgM and IgG antibodies in the serum for an early diagnosis of the disease often yields nonspecific findings, is time consuming and impractical [12, 13]. Liver biopsy is the most reliable method for diagnosing HSV hepatitis. Although transvenous biopsy is preferred over percutaneous biopsy, there remains a risk of bleeding with this method in patients with liver failure [12, 14–18]. Therefore, a PCR test for HSV-DNA is recommended in these patients [12, 14, 16, 17]. In our case, liver biopsy was difficult to perform due to the patient’s general condition; however, a diagnosis was made on the basis of the skin lesions and results of immunological tests. The level of anti-HSV-IgM antibody was elevated, and HSV-DNA was detected by PCR. Although we were unable to perform a liver biopsy, there were no other factors that could have caused liver dysfunction, hence the diagnosis of HSV hepatitis.

ACV is a systemic treatment for HSV hepatitis. Since early administration of ACV improves the prognosis, empirical administration of ACV is recommended in conditions where the cause of hepatitis is unknown [12, 15]. It has been reported that a delay of 36–48 hours in the administration of ACV may significantly influence the course of the disease [19]. Additionally, early therapy with ACV may result in a survival rate of 69% [20]. In this case, the diagnosis was delayed, as skin lesions appeared only during hospitalization, and it took time for blood tests to confirm the presence of HSV. Therefore, the start of treatment was delayed. In one study, the rate of death or need for liver transplantation was 51% in the acyclovir-treated group, and 88% in the nontreated group, and it was recommended that patients with acute liver failure of unknown etiology be treated with ACV until HSV hepatitis is ruled out [10]. In this case, PE and HDF improved the level of consciousness and coagulopathy, and both were discontinued once because of the improvement in blood tests. Once skin lesions appeared and blood tests confirmed HSV hepatitis, ACV was initiated. Although ACV was not administered before the diagnosis, HSV hepatitis could have still been treated; hence, death was thought to have been due to bacteremia. The patient was immunocompromised due to significant liver damage and steroid administration, and persistent bacteremia may have directly contributed to his death. Although levels of hepatobiliary enzymes increased prior to death, these improved after ACV administration. However, the worsening of the patient’s condition was considered to be caused by drug-induced liver injury of the congestive type due to ACV, which is different from acute liver failure caused by HSV hepatitis.

In conclusion, HSV hepatitis has a high mortality rate and should be treated early with ACV; however, the disease is difficult to diagnose. Acute liver failure can lead to a variety of complications that can result in death. Thus, careful observation is necessary, even after the resolution of acute liver failure.

## Data Availability

The datasets generated during and/or analyzed during the current study are available from the corresponding author on reasonable request.

## Abbreviations

ACV: Acyclovir
PCR: Polymerase chain reaction
ALF: Acute liver failure
HSV: Herpes simplex virus
PE: Plasma exchange
HDF: Hemodiafiltration
AST: Aspartate aminotransferase
ALT: Alanine aminotransferase
LDH: Lactate dehydrogenase
WBC: White blood cell
PT: Prothrombin time
PT%: Percentage of prothrombin
PT–INR: Prothrombin time–international normalized ratio
APTT: Activated partial thromboplastin time
